# Clinical significance of risk factor analysis in pancreatic cancer by using supervised model of machine learning

**DOI:** 10.3389/fmed.2025.1551926

**Published:** 2025-05-26

**Authors:** Amir Sherchan, Feng Jin, Bhakti Sherchan, Sujit Kumar Mandal, Binit Upadhaya Regmi, Ranita Ghising, Sandesh Raj Upadhaya, Bishnu Gautam, Dipendra Pathak, Maoquan Li

**Affiliations:** ^1^Department of Interventional and Vascular Surgery, Shanghai Tenth People’s Hospital, School of Medicine, Tongji University, Shanghai, China; ^2^Department of General Surgery, Scheer Memorial Adventist Hospital, Kavre, Nepal; ^3^District Hospital, Doti, Nepal; ^4^Patan Hospital, Patan Academy of Health Sciences, Kathmandu, Nepal; ^5^Trishuli Hospital, Nuwakot, Nepal; ^6^Department of Radiology, Buddha International Hospital, Dang, Nepal; ^7^Department of Radiology, Shanghai Pulmonary Hospital, School of Medicine, Tongji University, Shanghai, China

**Keywords:** pancreatic cancer, risk factors, risk scoring, machine learning, supervised model

## Abstract

**Introduction:**

Pancreatic cancer (PC) poses a significant global health challenge due to its aggressive nature, late-stage diagnosis, and high mortality despite advancements in treatment. Early detection remains crucial for timely intervention. This study aimed to identify clinically relevant predictors of pancreatic cancer using a supervised machine learning approach and to develop a risk stratification tool with diagnostic capabilities.

**Methods:**

A matched case-control study was conducted retrospectively at the Tenth People’s Hospital of Tongji University (2017–2023), involving 353 cases and 370 matched controls. Demographic and hematological data were extracted from medical records. Variables were pre-selected using cluster dendrograms and subsequently refined using logistic regression with backward elimination and Support Vector Machine (SVM) models. A final risk scoring model was developed based on the best-performing model and internally validated.

**Results:**

Key predictors retained in the final logistic regression model included Hemoglobin A1c (HbA1c) (OR 1.28; 95% CI: 1.08–1.52), Alkaline Phosphatase (ALP) (OR 1.02; 95% CI: 1.01–1.03), CA19-9 (OR 1.01; 95% CI: 1.01–1.01), Carcinoembryonic Antigen (CEA) (OR 1.41; 95% CI: 1.20–1.66), and Body Mass Index (BMI) (OR 0.88; 95% CI: 0.81–0.97). The final model demonstrated excellent diagnostic performance (AUC = 0.969, *p* < 0.001), with high accuracy, sensitivity, and specificity. A nomogram was constructed to facilitate individualized PC risk assessment.

**Conclusion:**

HbA1c, ALP, CA19-9, CEA, and BMI were independently associated with pancreatic cancer. The machine learning-derived risk scoring model demonstrated high predictive accuracy and may serve as a valuable clinical tool for early detection and screening of pancreatic cancer.

## 1 Introduction

Cancer continues to pose a significant global public health concern due to its exceptionally high mortality rate, despite several advanced therapeutic approaches (1). Pancreatic cancer (PC) among all malignant tumors has the highest mortality rate with an aggressive behavior and a poor prognosis (2, 3). Recently, both in men and women, primarily among older adults but increasingly in younger populations, pancreatic cancer (PC) has risen in incidence with a 5-year survival rate of only 10% (4). According to Global Cancer Statistics 2022, PC ranks 12th in incidence and 6th in cancer-related mortality worldwide (5). Based on Cancer Statistics 2021, the American Cancer Society reported approximately 60,430 new cases and 48,220 deaths for PC in the United States; ranking as the third deadliest cancer after lung/bronchus and colorectal cancers (6). Currently, the number of deaths from pancreatic cancer (PC) is increasing and it is predicted to be the second leading cause of cancer deaths in the U.S by 2030 (7). Over the last 2 decades, pancreatic cancer (PC) incidence has risen steadily, accounting for **∼** 2% of all cancers and 5% of cancer-related deaths (8). In China, pancreatic cancer (PC) incidence ranks 10th and 6th in mortality among malignant tumors. These numbers were expected to grow in the upcoming years as a result of changes in lifestyle and an aging population.

As of now, the main treatment of pancreatic cancer (PC) is surgical resection for potential recovery. Despite this, pancreatic cancer (PC) is a covert illness that presents with non-specific symptoms. The majority of patients are found to be suffering from a late-stage illness, which suggests that receiving surgical treatment is not a feasible option (9). Therefore, there remains a critical need for reliable diagnostic approaches that can enhance the early detection of pancreatic ductal adenocarcinoma, particularly at a stage when surgical resection is still possible (10, 11). Research has demonstrated that patients with early stage pancreatic cancer without metastases have a 5-year survival rate of 29%, however patients with distant metastases have just 2.6% survival rate (12). Thus, identifying those who are at risk for various stages of pancreatic cancer is crucial for the analysis and early treatment of pancreatic malignant growth.

Recent epidemiological studies have focused on identifying those at higher risk, estimating the risk, and learning more about the symptoms of pancreatic cancer in order to enhance early identification. Known risk factors include advanced age, diabetes mellitus, gallbladder disease, and chronic pancreatitis (13). Weight loss, hyperglycemia, back pain, epigastric pain, and gastrointestinal issues are among the symptoms (14, 15). Limited research has investigated the factors influencing pancreatic cancer in relation to clinical signs and biochemical indicators (16, 17). However, in clinical practice, we frequently use a few biochemical indicators to comprehensively evaluate the illness. Related clinical signs with specific hematological indicators are essential for the early disease detection, timely therapeutic intervention, and an improvement in prognostic outcomes.

In recent years, we have thoroughly investigated in hematological examination for all suspected cases of malignant tumors, which finally relies on more widely utilized imaging techniques such as Computed Tomography, Ultrasonography, Magnetic Resonance Imaging, and pathological biopsy for final diagnosis. It may be greatly impacted by its financial factor. To address this problem, we have considered many hematological examinations with medical and family history for screening to identify relevant risk factors associated with pancreatic cancer. Several studies previously conducted in Korea have reported that not only the DM but also the elevated fasting blood glucose levels are associated with an increased risk of pancreatic cancer even if the levels are lower than the diagnostic threshold for DM (9, 18).

Alkaline phosphatase (ALP), a homodimeric enzyme, has a key role to remove phosphate groups. All tissues and organs express ALP; however, the liver, bile duct, kidney, and bones have the highest concentrations. Studies have demonstrated that elevated serum ALP levels over the years are significantly associated with a poorer prognosis in several cancers, including prostate (19–21), colorectal (22), triple-negative breast (23), nasopharyngeal (24), and esophageal cancer (25). However, there has never been a thorough discussion of the relationship between PC survival and serum ALP measurements made at significant times, particularly upon diagnosis or prior to or following curative resection. Furthermore, because PC patients’ ALP readings will unavoidably fluctuate throughout the course of the survival period, dynamic survival models that account for this time-dependent variability of ALP should also be employed to produce a more reliable conclusion. ALP level may be a sensitive biomarker of tumor proliferation because a previously published study indicated that in patients with resected esophageal cancer, higher ALP was significantly linked with lymph node involvement (26). It is plausible that an elevated ALP in patients with PC, particularly those who have had their pancreatic cancer (PC) removed, may be linked to lymph node involvement as another kind of solid malignant tumor. This could lead to an early recurrence and further advancement of the illness.

Similarly, in 1965, tissue from fetal colon and colon cancer was used to identify a glycoprotein known as carcinoembryonic antigen (CEA), which has a molecular weight of 180–200 kDa (27). In addition to colorectal cancer, CEA levels also rise in several other cancer types as well, such as thyroid, lung, and breast cancers (28–30). Furthermore, 30 to 60% of individuals with pancreatic cancer had elevated serum levels of CEA (31). The most widely used biomarker, CA19-9, is now thought to represent the best quality level for pancreatic cancer. As demonstrated in Luo et al. (32, 33), CA19-9 was employed in PC as a diagnostic marker, prognostic indicator, and therapeutic monitoring tool. In this way, it may be helpful to look at the factors linked to the risk of pancreatic neoplastic growth in relation to clinical symptoms in order to determine diagnostic criteria for clinical assessment.

The goal of machine learning (ML), a subset of AI, is to enable computers to learn from experience. To complete tasks, it uses algorithms that rely on large amounts of data (34). Prediction in modern medicine is challenging due to the abundance of data. Big data integration both observed and predicted is where machine learning shines in a non-linear, clever way (35). A recent study highlights the growing role of ML in oncology, showing its effectiveness in analyzing complex clinical datasets for improved cancer risk stratification (36). Broadly speaking, machine learning strategies are divided into four categories: semi-supervised, supervised, unsupervised, and reinforcement learning. Another technique to integrate different algorithms is grouping learning. ML classifiers are organized with the use of ROC curves, which show classifier performance. Plotting sensitivity against (1-Specificity), they are line graphs. Performance is indicated by the area under the ROC curve (AUC), where higher AUC values correspond to better performance. When assessing machine learning processes, additional measures including accuracy, sensitivity, specificity, R-squared value, Brier score, PPV, and NPV are frequently employed (37).

In order to identify risk factors for pancreatic cancer (PC) and use them to develop a risk assessment scale, we conducted a matched case-control study to retrospectively analyze the medical records of 353 pancreatic cancer (PC) patients and 370 control individuals at the Tenth’s People Hospital of Tongji University from January 2017 to December 2023. The goal of this study was to enable early detection and prompt treatment of pancreatic cancer (PC) patients in clinical practice.

## 2 Materials and methods

### 2.1 Study population

This study included patients who underwent pathological or imaging exams, or had clinical signs suggestive of pancreatic cancer (PC) at the Tenth People’s Hospital of Tongji University between January 2017 and December 2023. Out of 368 patients who met the primary screening criteria, 15 patients with incomplete data were excluded. Consequently, 353 patients were available at the final follow-up and included in the study. Similarly, a control group of 370 fracture patients admitted to the orthopedic department, matched by gender and age at the same hospital, was randomly selected during the same period.

### 2.2 Inclusion exclusion criteria

#### 2.2.1 Inclusion criteria

1. All patients who met primary screening criteria are included.
2. Blood tests of first visit after pancreatic cancer diagnosis prior to start of treatment.
3. Age more than 18 years are included.

#### 2.2.2 Exclusion criteria

1. Pancreatic cancers patients associated with others malignant tumor were not included.
2. Patients with incomplete data information were excluded.
3. Ages less than 18 year are excluded.

### 2.3 Study design

**FIGURE 1:**
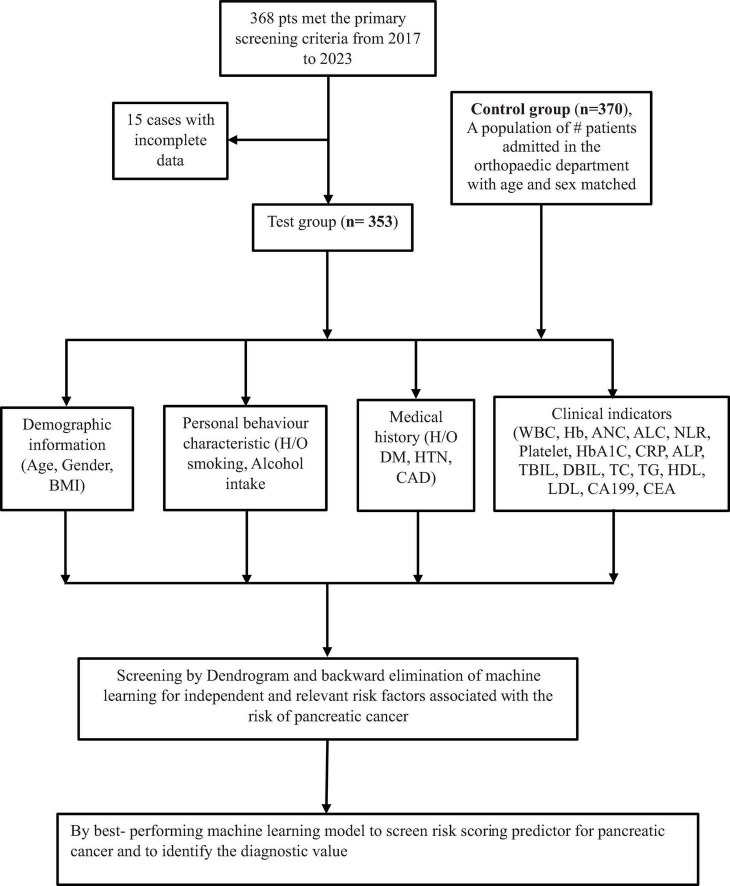
Study flow chart.

The following data were retrospectively collected through medical record reviews and telephone follow-ups: Demographics and history: age, gender, height, weight, body mass index (BMI), smoking history, alcohol consumption history, history of diabetes, history of hypertension, CAD, and laboratory parameters; lipid indexes: total cholesterol (TC), triglycerides (TG), high-density lipoprotein cholesterol (HDL-C), low-density lipoprotein cholesterol (LDL-C), WBC, ANC, ALC, Platelet, Hemoglobin, NLR, CRP, HbA1c, LFT indexes: ALP, DBIL, TBIL and tumor marker: carbohydrate antigen (CA19-9) and CEA levels. The factors associated with pancreatic cancer (PC) risk were first analyzed using a dendrogram for variable selection. Furthermore, variables selections were performed using machine learning of logistic regression with backward elimination and SVM under the Akaike information criterion (AIC) and feature importance ranking. Then a risk scoring population for pancreatic cancer were derived from best-performing model and also evaluate its diagnostic accuracy. The specific flow chart of the methodology is shown in Figure 1.

### 2.4 Statistical analysis

Data were entered in MS Excel^®^ and imported to R version 4.3.2 for data cleaning and analysis. Participant characteristics were described using numbers (percentages) for categorical variables and median (Interquartile range) for continuous variables. The distribution of predictor variables among pancreatic cancer and non-pancreatic cancer groups was compared using the Chi-square test and Wilcoxon rank sum test.

In the variables selection process, cluster dendrograms were constructed initially using the Hmisc package; employing Hoeffding’s distance for continuous variables and comparison of proportions for categorical variables. Variables were selected from the dendrogram based on their grouping within distinct clusters, indicating similarity (with a threshold of 30 times Hoeffding distance > 0.3), along with expert knowledge. Three continuous variables were removed from the dendrogram, whereas no categorical variable was removed from the dendrogram as the proportion of concurrent categories was lower than 0.25 for all the categorical variables. Furthermore, two distinct machine learning models were employed: Support Vector Machine (SVM) using e1071 package, and logistic regression models with backward elimination using rms package, aimed at identifying the most robust predictors of pancreatic cancer. Variables were further selected based on their importance derived from Akaike Information Criterion in logistic regression, and weights assigned by linear kernel SVM. Subsequently, the selected variables were used to execute the corresponding models, and their performance was assessed based on the receiver operating characteristic curve’s area under the curve (AUC). The logistic regression model exhibited the highest AUC among the two, thus chosen as the final model.

Odds ratios and their corresponding 95% confidence intervals (CIs) were calculated, with a significance level set at *p* < 0.05 (two-tailed). Internal validation of the final model was conducted using bootstrapping with 150 repetitions. Predictive performance of the model was evaluated through calibration and discrimination. Calibration was assessed by plotting observed proportions against predicted probabilities and a smoothed plot was obtained. Discrimination, indicating the model’s ability to differentiate between participants experiencing or not experiencing an event, was measured using the area under the receiver operating characteristic curve or c-statistics (ranging from 0.5 for chance to 1 for perfect discrimination) using the final logistic regression model, a nomogram was developed to predict the risk of pancreatic cancer using rms package.

## 3 Results

### 3.1 Basic information of the study participants

This study included 353 pancreatic cancer (PC) (case group) patients with a median age at onset of [68.0 years (63.0, 75.0)]. In the case group, there were 210 (59.5%) males and 143 (40.5%) females, for a male to female sex ratio of 1.46:1. In addition, 370 non-pancreatic cancer (control group) were selected from the fracture patients during the same period, with a median onset age of [68.0 years (62.0, 74.0)]. There were 165 (44.6%) males and 205 (55.4%) females in the control group for a male-to-female ratio of 1:1.24. Regarding age, there were no significant differences between the two groups whereas in gender there was significant difference (*p* = 0.6, *p* = < 0.001).

Regarding diabetes mellitus and smoking history, the proportion of patients in the case group was significantly higher than that in the control group (43.6% *vs.* 22.4%, *p* < 0.001, 27.8% *vs.* 17.3%, < 0.001). The proportion of hypertension and coronary artery disease history in the two groups were not statistically different whereas alcohol history in proportion was less significant in comparison to two groups (*p* = 0.8, *p* = > 0.9 and *p* = 0.033). The case group’s median BMI, Hb, ALC, TC and LDL-C levels were lower than that of the control group (*p* < 0.001). As shown in Table 1, the case group demonstrated statistically significant higher median levels of NLR, CRP, HbA1c, DBIL, TBIL, ALP, CA19-9 and CEA compared to the control group (*p* < 0.001); however, some continuous variables such as WBC, platelet, CRP, ANC and TG did not show statistically significant difference between two groups (*p* = 0.4, 0.3, 0.009, 0.7, and 0.2).

**TABLE 1:** Baseline characteristics of the study participants.

Predictors	Overall, *n* = 723^a^	Control, *n* = 370^a^	Case, *n* = 353^a^	*p*-value^b^
Gender [*n*, (%)]				<0.001
Male	375 (51.9)	165 (44.6)	210 (59.5)	
Female	348 (48.1)	205 (55.4)	143 (40.5)	
Age (years)	68.0 (62.0, 75.0)	68.0 (62.0, 74.0)	68.0 (63.0, 75.0)	0.6
BMI (kg/m^2^)	22.8 (20.7, 24.8)	23.5 (21.3, 25.8)	22.2 (20.0, 24.1)	<0.001
Smoking [*n*, (%)]				<0.001
Yes	162 (22.4)	64 (17.3)	98 (27.8)	
No	561 (77.6)	306 (82.7)	255 (72.2)	
Alcohol [*n*, (%)]				0.033
Yes	158 (21.9)	69 (18.6)	89 (25.2)	
No	565 (78.1)	301 (81.4)	264 (74.8)	
Diabetes mellitus [*n*, (%)]				<0.001
Yes	237 (32.8)	83 (22.4)	154 (43.6)	
No	486 (67.2)	287 (77.6)	199 (56.4)	
Hypertension [*n*, (%)]				0.8
Yes	326 (45.1)	165 (44.6)	161 (45.6)	
No	397 (54.9)	205 (55.4)	192 (54.4)	
Coronary artery disease [*n*, (%)]				> 0.9
Yes	87 (12.0)	45 (12.2)	42 (11.9)	
No	636 (88.0)	325 (87.8)	311 (88.1)	
White blood cell (10^9^/L)	6.5 (5.3, 8.2)	6.6 (5.4, 8.1)	6.5 (5.2, 8.2)	0.4
Hemoglobin	127.0 (114.0, 138.5)	131.5 (119.0, 142.8)	122.0 (109.0, 134.0)	<0.001
Lymphocyte	1.4 (1.0, 1.8)	1.5 (1.1, 2.0)	1.3 (0.9, 1.7)	<0.001
Platelet	210.0 (169.5, 260.0)	215.0 (174.0, 258.0)	207.0 (162.0, 262.0)	0.3
Neutrophil to lymphocyte ratio	3.1 (2.0, 4.9)	2.8 (1.9, 4.2)	3.4 (2.2, 5.6)	<0.001
C-reactive protein	6.1 (3.3, 18.9)	4.4 (3.3, 14.1)	8.3 (3.3, 22.8)	0.009
Hemoglobin A1c	6.1 (5.6, 6.9)	5.9 (5.5, 6.5)	6.3 (5.8, 8.3)	<0.001
Direct bilirubin (mmol/L)	5.1 (3.6, 8.0)	4.7 (3.6, 6.0)	5.7 (3.7, 63.2)	<0.001
Total bilirubin (mmol/L)	14.6 (10.7, 23.4)	14.1 (10.5, 18.8)	15.5 (10.7, 85.6)	<0.001
Neutrophil	4.3 (3.3, 5.9)	4.3 (3.3, 5.9)	4.3 (3.3, 5.8)	0.7
Total cholesterol (mmol/L)	4.3 (3.7, 5.0)	4.4 (3.9, 5.0)	4.1 (3.5, 4.8)	<0.001
Alkaline phosphatase	82.0 (67.5, 132.4)	72.2 (60.9, 82.3)	127.3 (81.8, 297.4)	<0.001
Triglyceride (mmol/L)	1.2 (0.9, 1.7)	1.1 (0.9, 1.6)	1.3 (0.9, 1.7)	0.048
HDL-C (mmol/L)	1.1 (0.9, 1.3)	1.1 (1.0, 1.3)	1.1 (0.8, 1.4)	0.2
LDL-C (mmol/L)	2.6 (2.0, 3.1)	2.7 (2.2, 3.2)	2.3 (1.8, 3.0)	<0.001
CA19-9 (U/mL)	19.0 (6.8, 435.7)	7.7 (4.8, 12.8)	436.3 (84.0, 1,000.0)	<0.001
CEA (U/mL)	2.5 (1.3, 5.2)	1.5 (0.8, 2.3)	5.2 (2.9, 11.8)	<0.001

^a^*n* (%); Median (IQR).

^b^Pearson’s Chi-squared test; Wilcoxon rank sum test.

### 3.2 Variables selection for risk of pancreatic cancer by dendrogram cluster analysis for both categorical and continuous predictors

**FIGURE 2:**
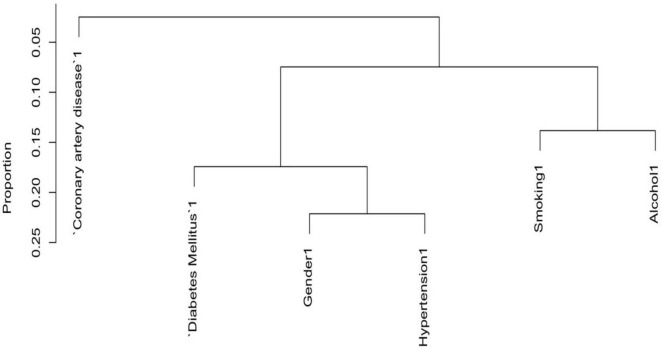
Cluster dendrogram for categorical variables.

**FIGURE 3:**
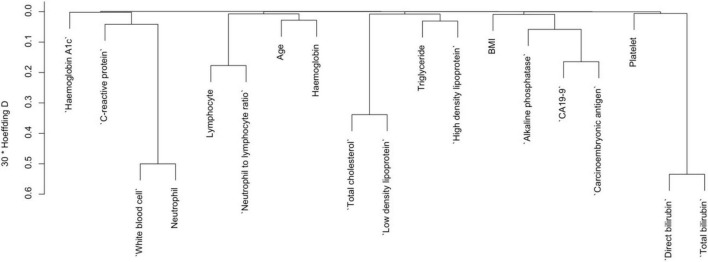
Cluster dendrogram for continuous variables.

To reduce the redundancy among predictor variables and to identify the distinct predictors of pancreatic cancer (PC), we used a cluster dendrogram. Figure 2 presents the cluster dendrogram of categorical variables. Since no variable exhibited a concurrent grouping of positive cases in more than 25% of observations, so we did not remove any variables. In Figure 3, three distinct clusters with high similarity indicated by > 0.3 Hoeffding’s distance, we removed three variables from the model based on expert knowledge (removed variables: Neutrophil, Total cholesterol, and Total bilirubin).

### 3.3 Further variable selection by backward elimination and features importance ranked

Since the outcome variable was binary, we employed and compared two classification methods to select the more parsimonious model for diagnosis of pancreatic cancer (PC). Using logistic regression and support vector machine (SVM), we ranked the importance of predictors for pancreatic cancer diagnosis. In the backward elimination process, the variables retained based on the AIC criteria were BMI, Hemoglobin A1c (HbA1c), Alkaline phosphatase, CA19-9, and Carcinoembryonic antigen. The top five most important variables for predicting pancreatic cancer in the case of SVM were CA19-9, Carcinoembryonic antigen, Alkaline phosphatase, neutrophil-to-lymphocyte ratio (NLR), and Hemoglobin A1c (HbA1c). With an AUC of 0.969 for the five predictors in the logistic regression model, we also retained the top five variables from SVM. However, the AUC of these variables in SVM was 0.906, indicating inferior performance compared to logistic regression. Therefore, we selected the logistic regression model for further development and internal validation, as presented in Table 2.

**TABLE 2:** Comparison of feature importance using different machine learning models.

Logistic regression with backward elimination			Support vector machine		
Predictors	*p*-value	AIC	Predictors	Feature weight	Importance Rank
**Variables deleted from the model**			**Variables kept**		
Alcohol	0.918	−1.99	CA19-9	3.56	1
Diabetes mellitus	0.975	−3.95	Carcinoembryonic antigen	3.12	2
Coronary artery disease	0.989	−5.88	Alkaline phosphatase	2.23	3
White blood cell	0.994	−7.78	Neutrophil to lymphocyte ratio	0.59	4
Platelet	0.994	−9.56	Hemoglobin A1c	0.37	5
Direct bilirubin	0.989	−11.09	**Variables discarded**		
C-reactive protein	0.986	−12.61	Direct bilirubin	0.33	6
Hypertension	0.978	−13.92	Smoking	0.33	7
Lymphocyte	0.957	−14.83	Age	0.2	8
Triglyceride	0.914	−15.37	Hemoglobin	0.17	9
High density lipoprotein	0.860	−15.81	Low density lipoprotein	0.16	10
Gender	0.767	−15.77	BMI	0.14	11
Neutrophil to lymphocyte ratio	0.664	−15.63	High density lipoprotein	0.14	12
Hemoglobin	0.528	−15.02	Alcohol	0.1	13
Low density lipoprotein	0.324	−13.09	Hypertension	0.1	14
Age	0.109	−8.84	Coronary artery disease	0.1	15
Smoking	0.0238	−3.63	Platelet	0.09	16
**Variables kept**			Gender	0.09	17
BMI			White blood cell	0.08	18
Hemoglobin A1c			Lymphocyte	0.08	19
Alkaline phosphatase			C-reactive protein	0.08	20
CA19-9			Diabetes mellitus	0.04	21
Carcinoembryonic antigen			Triglyceride	0.01	22

AUC for SVM: 0.906; AUC for LR: 0.969.

### 3.4 Odd ratio for final variables by logistic regression

The adjusted odds ratios (aORs) obtained from the logistic regression model are presented in Table 3.

**TABLE 3:** Odds ratio for final variables retained from backward elimination using logistic regression.

Variable	aOR (95% CI)	*p*-value
BMI	0.88 (0.81, 0.97)	<0.001
Hemoglobin A1c	1.28 (1.08, 1.52)	<0.001
Alkaline phosphatase	1.02 (1.01, 1.03)	<0.001
CA19-9	1.01 (1.01, 1.01)	<0.001
Carcinoembryonic antigen	1.41 (1.2, 1.66)	<0.001

aOR, adjusted odds ratio.

With a 1 kg/m^2^ increase in BMI, the odds of pancreatic cancer decreased by 12% (aOR: 0.88, 95% CI: 0.81, 0.97). Similarly, with a 1 unit rise in HbA1c, the odds of observing pancreatic cancer increased by 28% (aOR: 1.28, 95% CI: 1.08, 1.52).

Likewise, Alkaline phosphatase if increased by 1 unit, the odds for risk being pancreatic cancer accelerated by 2% (aOR: 1.02, 95% CI: 1.01.1.03). Subsequently, Common tumor marker CA199 if increased by 1 unit, then the odds of noting pancreatic cancer increased by 1% (aOR: 1.01, 95% CI: 1.01. 1.01). As such, most notably being CEA if increased by 1 unit, the odds of pancreatic cancer increased by 41% (aOR: 1.41, 95% CI: 1.2, 1.66) which indicates a significant role for increasing risk of pancreatic cancer.

### 3.5 Calibration plot with internal validation from logistic regression

**FIGURE 4:**
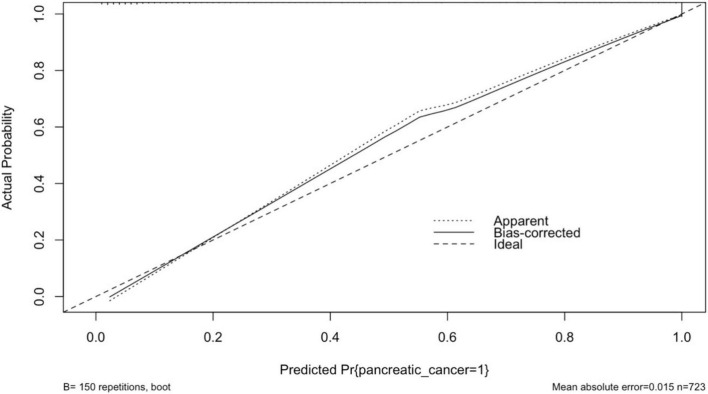
Calibration plot of the internal validation model from logistic regression.

The performance of the model was assessed using measures of calibration and discrimination. The results of calibration and description are presented in sections 3.5 and 3.6,” respectively. The smoothed calibration plot presented in Figure 4 indicates slight miscalibration in the 0.5 probability region, yet the bias-corrected probability has adjusted the curve toward the ideal line. Overall, the curve demonstrates acceptable calibration, with a mean absolute error of 0.015.

### 3.6 Final model performance

**FIGURE 5:**
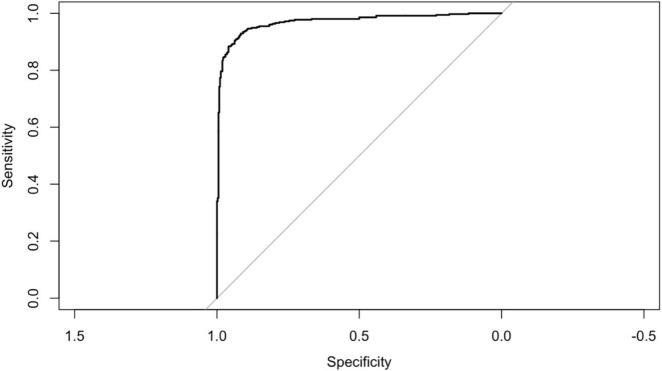
ROC curve for the final model from logistic regression.

Figure 5 displays the ROC curve for the final logistic regression model. The model demonstrates strong performance with an AUC of 0.969. Additionally, the accuracy is high at 0.9156 (95% CI: 0.8929, 0.9349), indicating the proportion of correctly classified cases. Sensitivity and specificity are also notable, with values of 0.9595 and 0.8697, respectively, highlighting the model’s ability to correctly identify positive and negative cases. Moreover, the positive predictive value (PPV) and negative predictive value (NPV) are 0.8853 and 0.9534, respectively, further indicating the model’s effectiveness in predicting outcomes. The balanced accuracy, reflecting the average of sensitivity and specificity, is also strong at 0.9146. Additionally, the *R*-squared value of 0.798 suggests that the model explains a substantial portion of the variance in the data. The Brier score of 0.062 indicates good calibration of the model’s predicted probabilities with observed outcomes.

### 3.7 Points predictor in nomogram for pancreatic cancer

**FIGURE 6:**
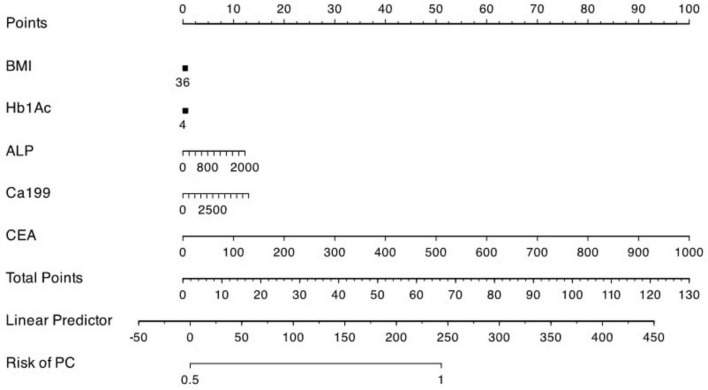
Nomogram of the model from logistic regression.

The result of the prediction model has been presented as a nomogram (Figure 6) for ease of interpretation and ease of use in clinical setting. Table 4 presents the points assigned to predictors in the nomogram for predicting pancreatic cancer. Each predictor, including BMI, HbA1c, ALP, CA19-9, and CEA, is associated with a specific point value based on its respective range. For instance, BMI ranges from 12 to 36, with corresponding points assigned accordingly, at 1 from 12 to 22 and 0 from 24 to 36, HbA1c ranges from 4 to 17, with corresponding points allocated accordingly; at 0 from 4 to 10 and 1 from 11 to 17, ALP ranges from 0 to 2,000, with corresponding points appointed accordingly; at 0 for 0 and 1 for 200, 2 for 400, 4 for 600, 5 for 800, 6 for 1,000, 7 for 1,200, 9 for 1,400, 10 for 1,600, 11 for 1,800, and 12 for 2,000, CA19-9 ranges from 0 to 5,500, with corresponding points assigned accordingly; at 0 for 0, 1 for 500, 2 for 1,000, 4 for 1,500, 5 for 2,000, 6 for 2,500, 7 for 3,000, 8 for 3,500, 9 for 4,000, 11 for 4,500, 12 for 5,000, and 13 for 5,500 and finally CEA ranges from 0 to 1,000, with corresponding points assigned accordingly; at 0 for 0, 10 for 100, 20 for 200, 30 for 300, 40 for 400, 50 for 500, 60 for 600, 70 for 700, 80 for 800, 90 for 900, and 100 for 1,000, respectively.

**TABLE 4:** Points for predictors in the nomogram.

BMI	Points	HbA1c	Points	ALP	Points	CA19-9	Points	CEA	Points
12	1	4	0	0	0	0	0	0	0
14	1	5	0	200	1	500	1	100	10
16	1	6	0	400	2	1000	2	200	20
18	1	7	0	600	4	1500	4	300	30
20	1	8	0	800	5	2000	5	400	40
22	1	9	0	1000	6	2500	6	500	50
24	0	10	0	1200	7	3000	7	600	60
26	0	11	1	1400	9	3500	8	700	70
28	0	12	1	1600	10	4000	9	800	80
30	0	13	1	1800	11	4500	11	900	90
32	0	14	1	2000	12	5000	12	1000	100
34	0	15	1			5500	13		
36	0	16	1						
		17	1						

Points per unit of linear predictor: 0.264601; linear predictor units per point: 3.779275.

Points for each five predictors ranges from 0 to 100 where each corresponding predictors; BMI and HbA1c within 0–10, ALP and Ca19-9 within 0–20 and CEA from 0 to 100 if added together to give their respective total points which total from 0 to 130 for their corresponding risk of pancreatic cancer to be associated with risk scoring from −50 to 450 are to be predicted linearly for reflecting risk of pancreatic cancer from 0.5 to 1 in proportion on the basis as shown in Figure 6.

## 4 Discussion

The mortality rate of PC is high, and the early diagnosis of the disease is difficult. Imaging plays a critical role in diagnosis of PC. However, accurately distinguishing PC from other pancreatic lesions remains a major diagnostic challenge because the imaging finding of PC can overlap with wide range of conditions, including inflammatory conditions (acute and chronic mass-forming pancreatitis, autoimmune pancreatitis, and paraduodenal pancreatitis), pancreatic neuroendocrine tumors, solid pseudopapillary neoplasms and metastases. This overlap may lead to potential misdiagnosis or delays in diagnosis, ultimately postponing timely intervention (38–40). Recent advancements in imaging techniques, along with the incorporation of clinical context and biochemical markers, have shown promises in enhancing diagnostic accuracy. Thus, developing accurate clinical scoring models is crucial in diagnosing and differentiating PC from other pancreatic pathologies.

Currently, China lacks a thorough PC screening program (17, 41). Previous research has examined a limited number of risk variables for pancreatic cancer, in addition to clinical signs. Our examination inspected PC risk factors and clinical indicators to foster a total clinical PC risk group scoring. This scale was useful for early identification of PC patients in clinical settings, based on general influencing factors. We found that Hemoglobin A1c (odds ratio: 1.28, 95% confidence interval: 1.08, 1.52), Alkaline phosphatase (odds ratio: 1.02, 95% confidence interval: 1.01, 1.03), CA19-9 (odds ratio: 1.01, 95% confidence interval: 1.01, 1.01), and Carcinoembryonic antigen (odds ratio: 1.41, 95% confidence interval: 1.2, 1.66) were associated with an increased risk of PC, whereas Body Mass Index (odds ratio: 0.88, 95% confidence interval: 0.81, 0.97) was associated with a reduction in the risk of PC. Taking into consideration these findings, the clinical PC risk scoring scale was found to be well-fitted in the population that was being modeled. Furthermore, the scale shown strong predictive value when it was used for screening the clinical PC risks scoring population. The discovery that body mass index (BMI) is adversely related with the risk of pancreatic cancer is in line with the findings of a meta-analysis conducted by Larsson et al. (42), which came to the conclusion that overweight and obesity are inversely associated with the incidence of pancreatic cancer. However, there is still no agreement about the relationship between BMI and PC. It is quite probable that this is related to the complicated hormonal and metabolic processes that influence the development of cancer. Greater BMI levels were found to be associated with a higher risk of PC in research of Jacobs et al. (43). Moreover, high BMI and a trajectory toward adult obesity were found to be positively correlated with PC in a 15-year subsequent study by Arjani et al. (44), with the association being higher in obesity with early onset and the male population. Controlling obesity throughout the adult life period may help prevent PC. The case group in our study had a lower BMI than the control group. Simultaneously, the results of the multifaceted analysis showed that BMI levels below the normal range were associated with an increased risk of PC. Taking into account that this was a case-control study, and the majority of patients had advanced PC at the moment of clinical analysis. Patients with advanced PC commonly experienced substantial weight loss due to cachexia.

Recent years have seen a significant increase in the amount of attention paid to the connection between hemoglobin A1C and PC. According to the findings of an analysis of the research, the risk of PC was shown to be inversely related to the amount of hemoglobin A1C, with persons who had just been diagnosed with increased hemoglobin A1C having the greatest risk of cardiovascular disease. Older patients with increased glycated hemoglobin (new onset diabetes) have about an 8-fold higher risk of developing pancreatic cancer than the general population (45). A multiethnic cohort study also demonstrated that recent-onset diabetes is a manifestation of pancreatic cancer and if long-standing diabetes then it plays a role of risk of developing pancreatic cancer (46).

In this study, we found that hemoglobin A1C was associated with an increased risk of pancreatic cancer. However, the connection between the hemoglobin A1C variable and PC was not that much significant. This suggests that glycated hemoglobin A1C may be an early clinical manifestation of pancreatic cancer.

Similarly, ALP is produced in every tissue or organ, although it is mostly concentrated in the kidney, liver, bile duct, and bones. Patients with PC will always have different ALP readings from successive tests. ALP level may therefore be a sensitive indication of tumor growth, as evidenced by a previously published study that indicated a higher ALP was significantly linked with involvement of lymph nodes in patients with resected esophageal cancer (26). An elevated ALP has been linked to lymph node involvement in PC patients, particularly in those who had their PCs removed. ALP was found to be elevated and linked to a greater possibility of pancreatic cancer in our study. ALP may be a risk factor in clinical detection for an early stage since PC is diagnosed lately with metastases.

Tumor markers such as CA 19-9 and CEA hold significant importance in the diagnosis and prognosis of pancreatic cancer; however, their clinical application is limited by several practical issues. In particular, their cost and limited availability in resource-poor settings restrict their widespread use for early detection or routine monitoring. A meta-analysis reported the sensitivity and specificity of CA 19-9 to be 81 and 82.8%, respectively, while CEA showed a sensitivity of 44.2% and specificity of 84.8%. Importantly, both markers have limited positive predictive value (PPV) when used for screening in asymptomatic populations (47, 48). These limitations underscore the need for cautious interpretation of serum marker results and support the recommendation that CA 19-9 and CEA should not be used in isolation but rather in conjunction with imaging modalities and clinical evaluation, particularly in symptomatic patients or those being evaluated for resectability or treatment response.

According to the discoveries of our examination, CA19-9 was found to be positively correlated with the risk of PC, which showed that the utilization of CA19-9 as a diagnostic sign for PC is vital for some degree. Right now, the main serologic diagnostic marker that is perceived for PC is the CA19-9. In any case, inflammation, false positive in non-PC conditions, and misleading negatives in Lewis’ antigen-negative patients are factors that could impair the diagnostic specificity of CA19-9 (32, 49, 50). It is conceivable that the early identification of pancreatic cancer may be aided by the revelation of novel serological markers, which, when paired with CA19-9 and other tumor indicators, could be utilized to conduct the test (49, 50).

The most widely utilized tumor marker was carcinoembryonic antigen (CEA), which was first identified as a tumor serum biomarker by Gold and Freedman (27). Malignant tissue, particularly gastrointestinal carcinomas, benign diseases, and normal, healthy people can all have CEA. Despite having limited sensitivity and specificity, CEA showed a considerable increase in distant metastasis of colorectal cancer when compared to non-distant metastases (51). Additionally, 30–60% of PDAC patients had higher serum CEA levels (31, 52). A prior study found that patients with low CA19-9 had less frequent CEA expression than those with high CA19-9 tumors (*p* < 0.0001) (53). Given that PC is diagnosed late, at the metastatic stage, screening for PC may be more important. Lately, the primary emphasis of the study was to examine the relationship between PC and CEA. Out of the five risk factors that were discovered, CEA was determined to be the one that was most strongly linked with PC.

The identification of relevant predictors of pancreatic cancer risk was accomplished by the use of logistic regression with backward elimination in the experiment. According to Chari et al. (54) and Goonetilleke and Siriwardena (55), the predictors that have been discovered, which include body mass index (BMI), hemoglobin A1c, alkaline phosphatase, CA19-9, and carcinoembryonic antigen, are in agreement with the recognized risk factors and biomarkers that are related with pancreatic cancer. This discovery is in line with the findings of a number of previously published articles that have emphasized the diagnostic and prognostic usefulness of these biomarkers in pancreatic cancer (55, 56). In particular, CA19-9 has been subjected to a great deal of research and has been confirmed as a biomarker for pancreatic cancer. According to Chari et al. (54), increased levels of CA19-9 are related with the existence of the illness as well as its development. This similarity with previously published research lends credence to the conclusions of our study, which increases their validity. The methods of logistic regression with backward elimination and Support Vector Machine (SVM) were used in our research in order to uncover important predictors of pancreatic cancer. Important characteristics that contribute to the prediction of pancreatic cancer (PC) include key predictors such CA19-9, hemoglobin A1c, alkaline phosphatase, and carcinoembryonic antigen. In line with previous studies that have highlighted the relevance of these biomarkers in the diagnosis and prognosis of pancreatic cancer (57, 58), our conclusion is consistent with those findings.

It is possible to prevent developing PC by avoiding growing overweight throughout the adult year. This is one way to avoid developing PC. Those who had a body mass index (BMI) of lower than normal range were found to have a risk of PC that was 1.99 times greater than those who had a BMI level of 21.5–24.4 kg/m^2^ (ratio: 1.99, 95% confidence interval: 1.03–3.84) (59). This was the finding that was made among former smokers who had a BMI. For the purposes of our study, the group that served as the case had a body mass index (BMI) that was lower than the group that served as the control. Furthermore, the results of the research showed that a lower body mass index (BMI) was associated with a decreased risk of acquiring cancer. This was proven by the findings of the study. Clinical characteristics such as body mass index (BMI) and hemoglobin A1c were included in the prediction model in addition to biomarkers from the previous section. This all-encompassing approach is in line with the current trend in pancreatic cancer research, which places an emphasis on the significance of including many risk variables in order to conduct an accurate risk assessment (60). By including these clinical factors into the model, the predictive potential of the model is improved, and doctors are provided with a more comprehensive understanding of the pancreatic cancer risk associated with a person.

It has been proven that the model has great performance, as shown by high accuracy, sensitivity, and specificity, in addition to a calibration plot that has been effectively calibrated. These findings are in line with those that were discovered in earlier research that evaluated prediction models for pancreatic cancer (61). We found that the robust performance of the model showed that it might have potential value in clinical practice for risk prediction and early diagnosis of pancreatic cancer. This is a job that continues to be difficult to accomplish owing to the fact that pancreatic cancer often presents itself in a late stage.

A further confirmation of the significance of body mass index (BMI), hemoglobin A1c, alkaline phosphatase, CA19-9, and carcinoembryonic antigen as significant predictors of pancreatic cancer risk is provided by the odds ratios and the logistic regression analysis. Based on the results of previous investigation, the percentage of individuals in the case group who tested positive for CA19-9 was exactly 84.0 percent. The fact that this rate was shown to have a positive link with the risk of PC demonstrated that the use of CA19-9 as a diagnostic indication for PC is significant to a certain degree that can be considered significant. As of right now, the CA19-9 is the only serologic diagnostic marker that is recognized for the presence of colon cancer. There are a number of variables that have the potential to reduce the diagnostic specificity of CA19-9. Previous research conducted (62–64) has shown a correlation between the development of PC and obesity (BMI), diabetes (Hemoglobin A1c), and biomarker levels. These results are in line with those findings. By using a support vector machine (SVM) model in addition to logistic regression, we were able to determine that CA19-9, Carcinoembryonic antigen, and Alkaline phosphatase were the most significant predictors. According to Kim et al. (56) and Koopmann et al. (65), these findings are in agreement with the results of the logistic regression, and they provide more evidence that these biomarkers are significant in the process of predicting pancreatic cancer. The logistic regression model demonstrated excellent performance measures, such as high accuracy, sensitivity, and specificity, as well as a high area under the ROC curve (AUC). The findings of this study are equivalent to or even beyond those that were published in other research that evaluated prediction models for pancreatic cancer (60, 61). The calibration plot and the calibration slope both suggest that the model has been appropriately calibrated, which further enhances the model’s reliability.

When it comes to predicting PC, the logistic regression model exhibits great performance, with high levels of accuracy, sensitivity, and specificity. With an area under the curve (AUC) of 0.969, the discrimination ability is quite good. The model has a high sensitivity, but there is need for improvement in terms of its specificity in order to cut down on the number of false positives. In investigations that are equivalent to this one, Rahib et al. (66) and Siegel et al. (6) found that these performance measures are comparable to or even better than those reported in those studies. The high accuracy of your logistic regression model, which is 91.56%, and the area under the curve (0.969) are similar to those that have been reported in previous research. For example, Zhang et al. (67) conducted research that used machine learning to reach an area under the curve (AUC) of 0.97 for PC prediction. This finding exemplifies the potential of these approaches in this particular field.

With the nomogram that was produced as a result of our results, doctors now have a user-friendly tool at their disposal to evaluate the individual risk of PC based on the predictors that were found. According to Balachandran et al. (68), nomograms have become more popular in the field of cancer due to its capacity to include a wide range of risk variables and to provide personalized risk assessments. Therefore, this makes a contribution to this area by providing a nomogram that is user-friendly and particularly designed for the evaluation of pancreatic cancer risk, which in turn makes it easier for clinical decision-makers to make educated choices. According to García-Albéniz et al. (69) and Vickers et al. (70), this coincides with the trend in personalized medicine, which involves the use of risk prediction models to assist in clinical decision-making and patient care tasks. The nomogram that was created based on the logistic regression model offers doctors a user-friendly tool that allows them to assess the risk of pancreatic cancer in a person based on the biomarker levels and clinical features of that individual. The research adds to this by offering a well-calibrated and accurate tool for pancreatic cancer risk assessment. Nomograms have been increasingly employed in clinical practice for risk prediction and decision-making Balachandran et al. (68) This work contributes to this trend by giving a nomogram.

The results of the study are in line with Rahib et al. (66) and Molina-Montes et al. (71) research on PC risk prediction models. These findings emphasize the significance of biomarkers, clinical factors, and machine learning approaches in the process of enhancing diagnostic accuracy and risk assessment. On the other hand, the research makes a contribution by providing a comprehensive analysis of the significance of features, odds ratios, model performance, and nomogram generation. This, in turn, improves the comprehension of PC risk prediction models and their usefulness in clinical settings. The purpose of this presentation is to provide insightful information on the development and evaluation of predictive models for the assessment of pancreatic cancer risk. Our work makes a contribution to the development of personalized medicine and to the improvement of patient care in the setting of pancreatic cancer. This is accomplished via the incorporation of thorough analyses and the development of a nomogram that user-friendly. Through the creation of prediction models and nomograms that are based on biomarkers and clinical characteristics, this makes a significant contribution to the evaluation of the risk of pancreatic cancer. The reliability of our study’s findings, as well as their potential therapeutic value, is bolstered by the fact that they are consistent with research that has already been published and that models perform very well.

Our results are consistent with Chari et al. (54) and Kim et al. (56) that have been published in the past about the significance of body mass index (BMI), hemoglobin A1c, CA19-9, and carcinoembryonic antigen as predictors of the risk of developing pancreatic cancer. The robustness of the technique that was provided in our work is shown by the fact that the performance metrics of the predictive model are comparable to or even better to those that were published in studies that were comparable to ours (60, 61).

Identifying risk factors associated with the risk of PC using common general factors and clinical indicators can assist in construction clinical screening criteria for PC, which can support physicians to ascertain high risk group for the purpose of screening and categorization of such patients to follow up. So that it can facilitate for early detection of pancreatic cancer from the high-risk group, its clinical adoption depends on prospective validation (72, 73).

Based on these contributing key predictors, we developed a clinical PC high risk scoring tool, called nomogram which has an excellent predictive performance under a point-based scoring system to estimate the risk of PC. Each biomarker is assigned weighted points based on clinically significant threshold (e.g., CEA ≥ 100 = 10 points, CA19-9 ≥ 500 = 1 point), with cumulative score delineated to a probability of PC risk if 66 total points = 100% risk. The clinical utility targets high-risk patients such as patients with new-onset diabetes (> 50 years), chronic pancreatitis, unexplained weight loss + abdominal pain and incidental elevated biomarker (CA 19-9, CEA, ALP) which are likely developing of PC. While this nomogram demonstrates internal validation but require future prospective validation in broader cohorts to confirm its predictive accuracy in clinical practice.

For the purpose of finding a solution to these issues, researchers have investigated the ways in which machine learning models may assist in the detection of pancreatic cancer. Using a variety of machine learning techniques, such as support vector machines (SVMs), logistic regression (LR), and deep learning approaches, several studies have investigated the analysis of imaging data and biomarkers with the purpose of achieving a more accurate diagnosis. The outcomes of these studies are promising because they have the potential to assist in the identification of subtle patterns that may be indicative of pancreatic cancer and for the improvement of the accuracy of diagnosis.

All things considered, earlier research on the identification of pancreatic cancer has prepared the way for the creation of more effective and trustworthy diagnostic techniques. Through the use of cutting-edge imaging technology, unique biomarkers, and machine learning models, researchers are making significant progress in enhancing early detection rates, facilitating prompt intervention, and eventually enhancing patient outcomes in the treatment of pancreatic cancer.

In general, the results of the study are in agreement with the previous research that has been conducted on the subject of predicting the risk of pancreatic cancer and evaluating biomarkers. Our knowledge of pancreatic cancer risk assessment is advanced as a result of this work, which also offers a significant tool for clinical practice. The study extends our understanding by adding both proven biomarkers and clinical characteristics into the prediction model.

However, one notable limitation of our study is the absence of external validation using an independent cohort. Although our model demonstrated strong predictive performance within the internal dataset, its generalizability to real-word setting remains uncertain. External validation is crucial to confirm the applicability of the model across different clinical setting. Therefore, further studies should be conducted to evaluate the model in larger, multi-center cohort to ascertain its utility and reliability in routine clinical practice.

## 5 Conclusion

In this study, we illustrated a clinically PC risk score scale (nomogram) using some selected feature importance and backward elimination from common factors and routine hematological indicators that were simple way to identify and acquired by supervised machine learning. The findings of this work, taken as a whole, provide evidence that supervised machine learning models have the potential to enhance pancreatic cancer risk assessment by discovering new risk variables and building effective prediction tools. It was clinically helpful and had a lower screening cost. The scale, meanwhile, has a few shortcomings. For instance, certain characteristics could only be demonstrated to correlate with PC due to the case-control study that was performed; hence, future research was required to confirm the investigation of the causative association.

## Data Availability

The original contributions presented in the study are included in the article/supplementary material, further inquiries can be directed to the corresponding author.
